# Successful Therapeutic ERCP in a 99-Day-Old Child With Common Bile Duct Stones: A Case Report and Discussions on the Particularities of the ERCP in Children

**DOI:** 10.3389/fped.2020.00435

**Published:** 2020-07-28

**Authors:** Qifeng Lou, Jianliang Sun, Xiaofeng Zhang, Hongzhang Shen

**Affiliations:** ^1^Department of Gastroenterology, Affiliated Hangzhou First People's Hospital, Zhejiang University School of Medicine, Hangzhou, China; ^2^Department of Anesthesiology, Affiliated Hangzhou First People's Hospital, Zhejiang University School of Medicine, Hangzhou, China

**Keywords:** child, common bile duct (CBD) obstruction, experience, anesthesiology, ERCP (endoscopic retrograde cholangiopancreatography), particularities

## Abstract

**Backgroud:** At present, therapeutic endoscopic retrograde cholangiopancreatography (ERCP) has gradually been used in the diagnosis and treatment of biliary and pancreatic diseases in children, but reports on and the application of ERCP in children, especially in infants, are still highly limited.

**Case Presentation:** This case report describes a 99-day-old infant with choledocholithiasis who successfully underwent ERCP to relieve an obstruction. The infant developed obstructive jaundice during chemotherapy for a malignant tumor, and a routine blood examination showed signs of infection. Liver damage also occurred. B-ultrasound suggested stones in the lower segment of the common bile duct (CBD). After sufficient communication and preparation, biliary drainage was successfully carried out in the infant using adult duodenoscope (JF240) and related instruments with cooperation from the Department of Anesthesiology.

**Conclusions:** This paper preliminarily introduces our experience with adult duodenoscope in children, providing a good example for hospitals without a special duodenoscope for children to carry out pediatric ERCP. Clinicians should pay close attention to the need of careful use of ERCP in infants.

## Introduction

The superiority and safety of endoscopic retrograde cholangiopancreatography (ERCP) in the diagnosis and treatment of biliary and pancreatic diseases in children have been increasingly recognized (1). However, ERCP has certain special characteristics for children In addition, the endoscopes used in ERCP in most hospitals are designed for adult. Thus, the technical limitations and difficulty of this operation are greater in children than in adults. Approximately 3,000 therapeutic ERCP procedures are performed in our center every year. Of these, ~120 are performed in children. Recently, therapeutic ERCP was successfully performed in a 99-day-old infant. We report this case and introduce our experience with ERCP in children.

## Case Presentation

A 99-day-old boy was admitted to our hospital because of “abdominal B-ultrasound indicating gallbladder sludge for 2 months.” The infant was diagnosed with left nephroblastoma at birth. In the second cycle of chemotherapy, abdominal B-ultrasound indicated a low echo area in the gallbladder, and the diagnosis of gallbladder sludge was accordingly considered. The child exhibited jaundice and a progressive elevation in gamma-glutamyl transferase (γ-GT). He was transferred to our hospital for further treatment. The physical examination on admission revealed the following: temperature: 36.7°C, pulse rate: 154 times/min, respiratory rate: 36 times/min, blood pressure: 90/62 mmHg, percutaneous oxygen saturation: 97%, and weight: 7.5 kg. Mild jaundice was noted. He had no rash or blood spots on his skin. The heart and lung examinations were unremarkable. His abdomen was flat and non-tender and had no rebound tenderness. No mass was palpated. Murphy's sign was negative. The laboratory results revealed the following (Table 1): total bilirubin (TB) 73.4 μmol/L, conjugated bilirubin (CB): 55.9 μ/L, alanine aminotransferase (ALT): 84 U/L, aspartate aminotransferase (AST): 103 U/L, γ-GT: 2,189 U/L, alkaline phosphatase (ALP): 607 U/L, blood amylase: 3 U/L, white blood cells (WBCs): 10.9 × l0^9^/L, percentage of neutrophils: 8.9%, and percentage of lymphocytes: 81.5%. Blood coagulation function and routine urine and stool values were normal. B-ultrasound was repeated and showed that the diameter of the upper segment of the CBD was 0.8 cm. A hypoechoic mass was noted in the lower CBD. The CBD and intrahepatic bile duct were dilated.

**Table 1:** Changes in laboratory results before and after treatment.

**Laboratory Results**	**Before ERCP**	**After ERCP (72 h)**
TB (μmol/L)	73.4	28.3
CB (μ/L)	55.9	23.7
ALT (U/L)	84	33
AST (U/L)	103	31
γ-GT (U/L)	2,189	1,033
AKP (U/L)	607	201
Blood amylase (U/L)	3	3
WBC(No./L)	10.9 × l0^9^	9.8 × l0^9^
Neutrophils (%)	8.9	24.1
Lymphocytes (%)	81.5	62.3

**Figure 1:**
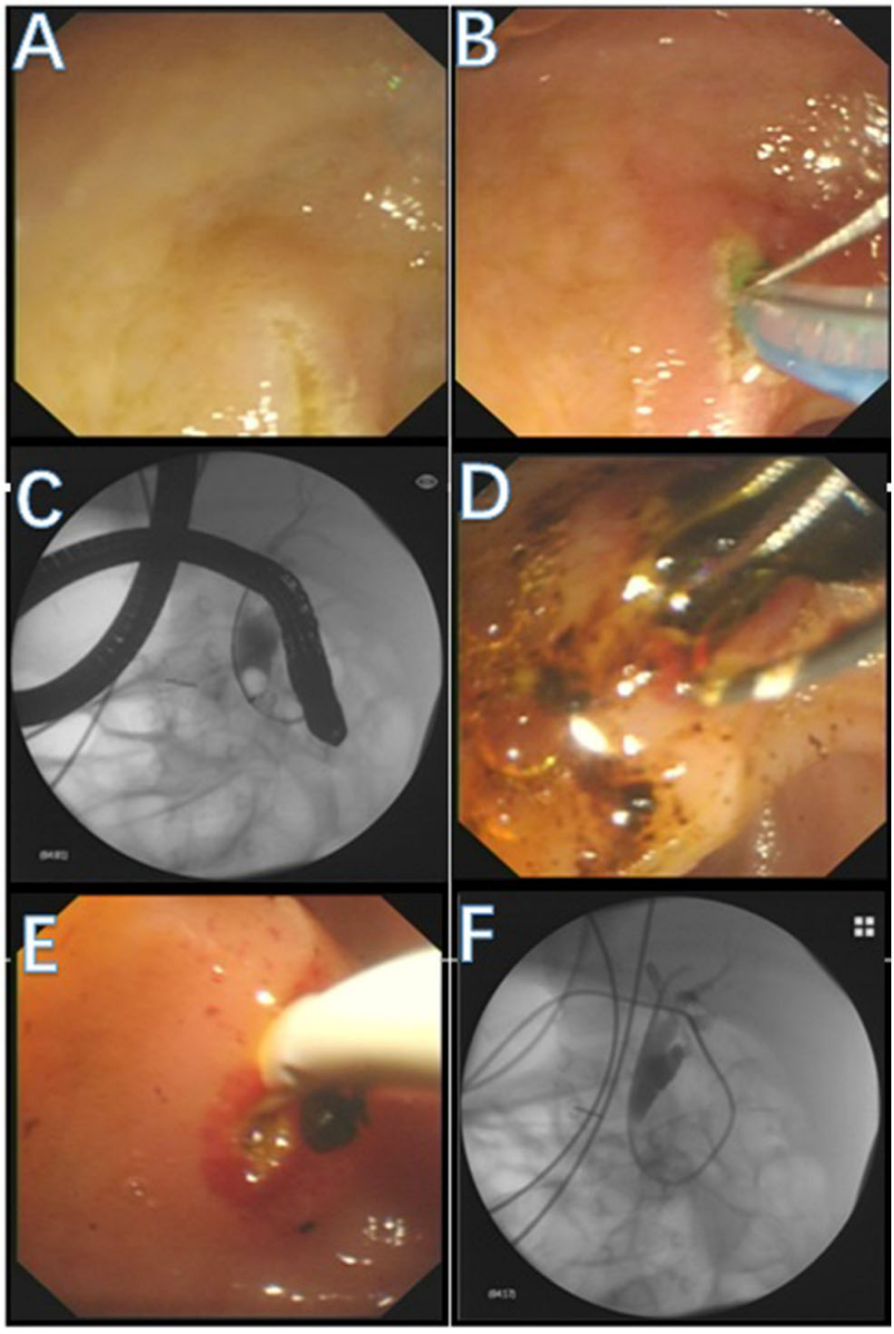
Operation process. **(A)** The duodenal papilla appeared. **(B)** An needle knife and a guide wire were used to achieve successful selective biliary cannulation. **(C)** Under fluoroscopy, cholangiography was performed and showed dilation of the CBD Multiple filling defects were observed in the CBD. **(D)** A grasping basket was used to remove multiple black stones. **(E)** A stone retrieval balloon was used to remove the stones in CBD. **(F)** A drainage catheter was placed at the end of the procedure.

After full communication and preoperative examinations were completed, the infant underwent ERCP. He was placed in the prone position and underwent ERCP under general anesthesia with intubation. An Olympus JF240 duodenal endoscope was used. The duodenal papilla appeared and had a granular, fluffy opening. An Olympus triple-lumen needle knife and a 0.025-inch Loach guide wire were used to achieve successful selective biliary cannulation. The bile sample obtained during backsuction was cloudy and dark. Under fluoroscopy, cholangiography was performed from the upper to lower segment of the CBD and showed dilation of the CBD (1.1 cm in diameter). Multiple contrast filling defects were observed in the CBD. At the 12 o'clock site of the bile duct, a small, 0.2 cm longitudinal incision was made in the papilla. No bleeding was observed at the cutting edge. A grasping basket was used to remove multiple black stones. Finally, a stone retrieval balloon was used to remove the stones in the bile duct. A 6 Fr straight-tip nasobiliary drainage catheter was placed at the end of the ERCP (Figures 1A–F). The endotracheal tube was removed upon awakening. The patient had stable vital signs and was returned to the ward. An laboratory results were repeated 72 h after ERCP and showed the following (Table 1): TB: 28.3 μmol/L, CB: 23.7 μmol/L, ALT: 33 U/L, AST: 31 U/L, γ-GT: 1,033 U/L, AKP: 201 U/L, blood amylase: 3 U/L, WBCs: 9.8 × l0^9^/L, neutrophils: 24.1%, and lymphocytes: 62.3%. The jaundice markedly improved. He was energetic and had acceptable food intake. Four days after ERCP, cholangiography was performed via a nasobiliary catheter and showed smooth draining of the contrast agent and no residual stones in the bile duct. Then, the nasobiliary catheter was removed. A liver function examination was repeated 1 week after ERCP; the profile had returned to normal. The patient was discharged.

## Discussion

### Advantages and Disadvantages of ERCP in Children

ERCP is an invasive procedure, and its application in children is still limited. It has been reported that ERCP in children accounts for 3.3% of all ERCP procedures (2) and ~4% in our center. The success of endoscopic treatment depends on a full evaluation of the condition before ERCP and the skill of the doctor. The indications for ERCP cannot be blindly extended, which may cause unnecessary complications (3). Before ERCP, it is necessary to fully inform the child's family members of the child's condition, and informed consent must be obtained regardless of which plan is adopted. At present, ERCP in children under 1 year old is mostly used for diagnosis (2, 4), but it is difficult to be used for treatment, and the success rate is lower than that of children in other age groups (4). If the child's family members choose to undergo therapeutic ERCP, it is important to ensure that they fully acknowledge the details of the procedure, the benefits to the child and the possible complications after ERCP. Second, the operation team should ensure that they have enough ERCP experience in children and can identify and address various complications in time to avoid endangering the child's life.

### Difficulties in Using ERCP in Children

ERCP has been applied in children since the mid-1970s. Although it has similar processes as adult ERCP, it has its own characteristics: children often cannot tolerate an ERCP and need sedation or anesthesia (5). The digestive lumen in children are relatively small, and the mucosa is very delicate. Duodenoscopy must be performed gently to avoid mucosa edema, bleeding and even perforation. However, although ERCP has been increasingly used in children, no dedicated pediatric duodenoscope is available in China and many other countries. In addition, Olympus has stopped producing pediatric duodenoscope (4). We used the JF240 duodenoscope (1.1 cm diameter, 3.2 mm working channel) for the procedure, which was designed for adult. The disadvantages are that this endoscope cannot easily pass through the throat of an infant and that the operation may affect breathing and require tracheal intubation. An endoscope 1.1 cm in diameter was inserted into the intestinal lumen, which may reduce the working space and increase the operation difficulty due to limited vision.

### Prevention of the ERCP Complications—Practical Tips

The success rate of ERCP (~90%) in children is similar to or slightly lower than that of adults (4). The older the age, the higher the success rate is (6), while the incidence of complications (0–10%) is similar to or slightly higher than that of adults (2, 4, 7). Such complications include perforation, bleeding and post-ERCP pancreatitis (PEP) (8). However, the digestive tract of children is immature, increasing the likelihood of perforation. Here are some key points: First, because the insertion part of endoscope is relatively large, the application of lidocaine jelly to the insertion part is recommended (9). It can reduce irritation and increase lubrication.

Second, the operator should have sufficient skill in duodenoscopy. Particular caution should be paid when passing the endoscope through the upper corner of the duodenum at an appropriate angle according to the anatomical shape of duodenum. The endoscopist should not pull back blindly; pushing the endoscope forward is more important. When the endoscope reaches the papilla, the papilla should be fully exposed and evaluated. According to the axial direction of the pancreatobiliary tract, selective biliary cannulation should be performed, and the curve of the three-lumen needle knife should also be adjusted. The papilla in children is very delicate. Therefore, controlling the force required to push the guide wire is especially important. It is important to distinguish the difference when the guide wire is in the bile duct, pancreatic duct (PD), submucosal layer or circular muscle. It is necessary to stop the operation if the guide wire enters the submucosal layer or circular muscle. If repeated cannulation fails, ERCP should be abandoned to avoid complications. With regard to the prevention of PEP, there is no conclusion that the use of rectal non-steroidal anti-inflammatory drugs is beneficial, and the placement of PD stents is still controversial. A large-scale study showed that the prophylactic placement of PD stents during ERCP in children could increase the incidence of PEP (10), while another study showed that the prophylactic placement of PD stents had a tendency to reduce PEP (2); therefore, this issue needs further research.

When cannulation is successful, experimental backsuction is necessary to obtain bile to confirm successful catheter placement. Bile can be used for bacterial culture. Moreover, backsuction can reduce the pressure in the bile duct. The injection of contrast agent into the bile duct should be gentle and at an even rate under fluoroscopy. Excessive force or a high injection rate should not be used because they can cause excessive pressure in the biliary tract, which can cause retrograde infection (11). Furthermore, an evaluation of the papilla is required before making an incision. The size of the incision at the papilla should be determined according to the size of the stones or the basket to ensure smooth basket placement and stone removal. Pediatric bile duct stones are generally composed of a mixture of biliary sludge, which can be removed by the stone basket after mechanical fragmentation. Therefore, a small incision is reasonable as long as the basket can smoothly enter the bile duct.

### Anesthesia for ERCP in Children

A conscious child cannot tolerate the ERCP operation. ERCP in children must be under proper sedation or anesthesia to reduce the risk of mucosal damage, bleeding and perforation (12). However, the current specific sedation or anesthesia methods for pediatric endoscopy remain controversial. Both deep sedation and general anesthesia with intubation can be safely used in children (13).

General anesthesia with intubation has the advantages of ensuring good ventilation and oxygenation. Due to relatively high airway resistance in children, they are prone to airway obstruction in any situation. In addition, the lateral position during ERCP can inhibit breathing and cause insufficient ventilation. For this infant, we used general anesthesia with intubation. The anesthetics were discontinued when the endoscope was withdrawn. Hand-controlled ventilation began to quickly expel sevoflurane. The tracheal tube was removed when the child awoke spontaneously and could respond to outside stimulation with eye opening. The child was transferred to a postoperative room for further observation. To reduce respiratory and oropharyngeal secretions in children and maintain a patent airway, atropine and low-dose dexamethasone can be routinely used before induction to inhibit glandular secretions; to prevent possible allergic reactions to the contrast agent, postoperative throat discomfort and edema; and to ensure the smoothness of the airway during and after ERCP.

In conclusion, ERCP is increasingly being widely used in children and should attract the attention of clinicians, especially endoscopists. We need more experience with adult duodenoscopy in children, and we hope that this technology will benefit more children. However, in terms of implementing pediatric ERCP, a sufficient risk assessment must be carried out, and informed consent must be obtained. The prevention of complications during ERCP, corresponding emergency plans and cooperation with anesthesia should be maintained to ensure the safety of ERCP in children. Otherwise, this procedure could have serious consequences.

## Data Availability Statement

All datasets generated for this study are included in the article/supplementary material.

## Ethics Statement

Written informed consent for ERCP in this infant was obtained from the patient's legal guardian. Written informed consent from the patient's legal guardian was obtained for the publication of this case report and accompanying images. Copies of the written consent form are available for review from the editors of this journal.

## Author Contributions

QL assisted in the ERCP procedure, drafted and revised the manuscript, and approved the final manuscript. JS completed the whole anesthesia process, drafted the manuscript, and approved the final manuscript. XZ conceived the study, identified the disease, performed most of the ERCP procedure, revised and reviewed the manuscript, and approved the final manuscript. HS conceived the study and assisted with the ERCP procedure, drafted and revised the manuscript, reviewed the manuscript, and approved the final manuscript. All authors contributed to the article and approved the submitted version.

## Conflict of Interest

The authors declare that the research was conducted in the absence of any commercial or financial relationships that could be construed as a potential conflict of interest.

## Abbreviations

TB: total bilirubin
CB: conjugated bilirubin
ALT: alanine aminotransferase
AST: aspartate aminotransferase
γ-GT: gamma-glutamyl transferase
AKP: alkaline phosphatase
WBC: white blood cells.
